# Human ABC and SLC Transporters: The Culprit Responsible for Unspecific PSMA-617 Uptake?

**DOI:** 10.3390/ph17040513

**Published:** 2024-04-16

**Authors:** Harun Taş, Gábor Bakos, Ulrike Bauder-Wüst, Martin Schäfer, Yvonne Remde, Mareike Roscher, Martina Benešová-Schäfer

**Affiliations:** 1 German Cancer Research Center (DKFZ), Research Group Molecular Biology of Systemic Radiotherapy, Im Neuenheimer Feld 280, 69120 Heidelberg, Germany; harun.tas@dkfz-heidelberg.de (H.T.); g.bakos@dkfz-heidelberg.de (G.B.); u.bauder-wuest@dkfz-heidelberg.de (U.B.-W.); 2 German Cancer Research Center (DKFZ), Service Unit for Radiopharmaceuticals and Preclinical Trials, Im Neuenheimer Feld 280, 69120 Heidelberg, Germany; martin.schaefer@dkfz-heidelberg.de (M.S.); y.remde@dkfz-heidelberg.de (Y.R.); mareike.roscher@dkfz-heidelberg.de (M.R.)

**Keywords:** targeted radionuclide therapy, targeted alpha therapy, PSMA, PSMA-617, prostate cancer, efflux transporters, uptake transporters, salivary gland toxicity, kidney toxicity, side-effects

## Abstract

[^177^Lu]Lu-PSMA-617 has recently been successfully approved by the FDA, the MHRA, Health Canada and the EMA as Pluvicto^®^. However, salivary gland (SG) and kidney toxicities account for its main dose-limiting side-effects, while its corresponding uptake and retention mechanisms still remain elusive. Recently, the presence of different ATP-binding cassette (ABC) transporters, such as human breast cancer resistance proteins (BCRP), multidrug resistance proteins (MDR1), multidrug-resistance-related proteins (MRP1, MRP4) and solute cassette (SLC) transporters, such as multidrug and toxin extrusion proteins (MATE1, MATE2-K), organic anion transporters (OAT1, OAT2v1, OAT3, OAT4) and peptide transporters (PEPT2), has been verified at different abundances in human SGs and kidneys. Therefore, our aim was to assess whether [^177^Lu]Lu-PSMA-617 and [^225^Ac]Ac-PSMA-617 are substrates of these ABC and SLC transporters. For in vitro studies, the novel isotopologue ([α,β-^3^H]Nal)Lu-PSMA-617 was used in cell lines or vesicles expressing the aforementioned human ABC and SLC transporters for inhibition and uptake studies, respectively. The corresponding probe substrates and reference inhibitors were used as controls. Our results indicate that [^177^Lu]Lu-PSMA-617 and [^225^Ac]Ac-PSMA-617 are neither inhibitors nor substrates of the examined transporters. Therefore, our results show that human ABC and SLC transporters play no central role in the uptake and retention of [^177^Lu]Lu-PSMA-617 and [^225^Ac]Ac-PSMA-617 in the SGs and kidneys nor in the observed toxicities.

## 1. Introduction

Prostate cancer (PCa) persists as a vicious cancer type amongst the male population worldwide. Currently, it ranks as the second most frequently diagnosed cancer type (14.1%) and fifth in terms of overall cancer mortality (6.8%) [1]. Today, localized PCa can be treated efficiently if diagnosed early; however, the treatment of the more advanced metastatic castration-resistant form (mCRPC) still bears significantly lower 5-year survival rates (15%) and calls for improved treatment regimens [2,3]. With the successful identification of the prostate-specific membrane antigen (PSMA), a frequently overexpressed target on the surface of PCa cells, the door to novel theranostic treatment modalities was opened [4,5,6]. Throughout the past decade, a high number of novel radioligands have emerged for the diagnosis and treatment of PCa, with [^177^Lu]Lu-PSMA-617 as the current gold standard in compassionate use all around the globe [7,8,9]. Most recently, [^177^Lu]Lu-PSMA-617 (Pluvicto^®^, Novartis—Basel, Switzerland) has become the very first PSMA-targeted radionuclide therapy (TNRT) against mCRPC to be approved by the Food and Drug Administration (FDA, USA), the Medicines and Healthcare products Regulatory Agency (MHRA, UK), Health Canada and the European Medical Agency (EMA, EC) [10,11].

The success of [^177^Lu]Lu-PSMA-617 is not without its shortcomings, however. Undesired uptake into healthy organs has been observed, as PSMA is not solely expressed on malignant PCa cell surfaces but also at basal levels in healthy, e.g., renal, lacrimal and salivary gland, tissues [12,13]. Previous studies on [^177^Lu]Lu-PSMA-617 have reported its high accumulation in the salivary glands (SGs, up to 1 Gy/GBq injected dose) [14] and kidneys (0.5–0.6 Gy/GBq injected dose) [15]. This undesired uptake causes severe xerostomia [16] and possibly renal dysfunction [15], much to the detriment of patients’ quality of life, not infrequently leading to treatment abandonment altogether [17]. Competition assays against non-radioactive PSMA ligands have revealed only partial displacement of the uptake in the SGs and kidneys, indicating non-specific uptake mechanisms, which might play a major role in the occurrence of side-effects [18,19,20]. Consequently, there is an urgent need to elucidate the underlying uptake in order to effectively counter undesirable ligand accumulation in healthy organs.

As many drugs are metabolized into their ionic counterparts, the ionic charge and molecular weight of PSMA-based ligands are hypothesized to factor into their toxic SG and kidney uptake [19,21]. The absorption, distribution, metabolism and excretion (ADME) processes of various drugs, such as xenobiotics, are handled through members of the substrate-specific ATP-binding cassette (ABC) and solute carrier (SLC) membrane transporter superfamilies [22], which are widely expressed in the liver, intestine, kidneys and blood–tissue barriers [23], with previous studies also hinting at their presence in the SGs [24,25].

**Figure 1:**
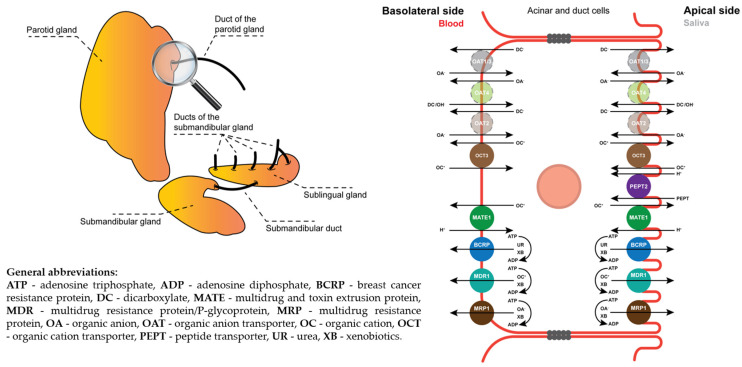
Schematic overview of the human SGs (**left**) and close-up of drug transporters located in their acinar and duct cells (**right**). Acinar and duct cells contain OAT1-4, OCT3, MATE1, BCRP, MDR1 and MRP1 transporters in the basolateral and apical membranes, with the exception of PEPT2, located in the apical membrane only. Note: Opaque coloring and dashed borders indicate transporters detected using IHC and qPCR experiments but not using MS/MS experiments.

According to Romanska et al., the abundance of ABC and SLC transporters in the SGs was concluded to be as follows: OCT3 (43%), MRP1 (31%), PEPT2 (10%), MRP4 (7%), MATE1 (5%) and BCRP (4%) [26] (Figure 1).

Within the ABC transporter family, the presence of P-glycoproteins (MDR1), MRP1 and MRP2 was revealed at both the mRNA [27,28] and protein levels, located in the basolateral and luminal membranes of the ductal SG cells [29,30]. Furthermore, immunohistochemistry (IHC) staining against SLC transporters revealed the expression of organic anion transporters (OAT1–4) in whole SGs [31] and the organic cation transporter OCT3 in the apical and basolateral membranes of SG acinar cells [32].

**Figure 2:**
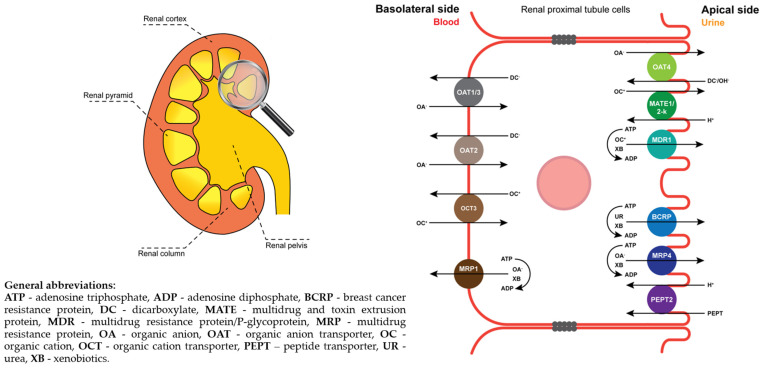
Schematic overview of the human kidney (**left**) and close-up of drug transporters located in renal proximal tubule cells (**right**). Renal proximal tubule cells contain OATs1-3 and MRP1 in the basolateral membrane and OAT4, MATE1, MATE2-K, MDR1, BCRP, MRP4 and PEPT2 in the apical membrane.

These and other transporters may also be involved in renal excretion processes [33], in the basolateral (OAT1, OAT3, OAT4PC1, OCT2) and in the apical (MDR1, BCRP, MATE1, MATE2-K, OAT4, MRP2, MRP4) membranes of the renal proximal tubules [34]. As compared to the SGs, the abundance of these transporters is different in the kidneys. The protein levels of OAT1, OCT2 and MATE1 have been revealed to be much higher in comparison to other vital transporters (OAT3, OAT4, MDR1, BCRP, MATE2-K, OATP4C1, MRDP and MRP4), as reported by Basil et al. [35] (Figure 2).

**Figure 3:**
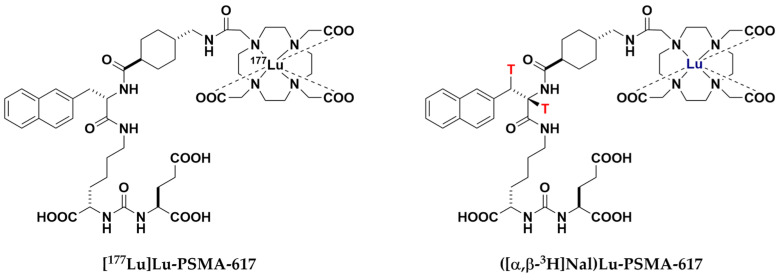
Chemical structure of [^177^Lu]Lu-PSMA-617 and its novel isotopologue ([α,β-^3^H]Nal)Lu-PSMA-617.

As regards these recent data, we examined whether human SLC and ABC transporters play a role in the efflux and uptake of [^177^Lu]Lu-PSMA-617 and [^225^Ac]Ac-PSMA-617 in the SGs and kidneys and their subsequent toxicity. In detail, ([α,β-^3^H]Nal)Lu-PSMA-617 [36], an isotopologue of [^177^Lu]Lu-PSMA-617 (Figure 3), was assessed as an in vitro inhibitor of the human ABC transporters BCRP, MDR1, MRP1 and MRP4 and as an in vitro substrate of the human SLC transporters MATE1, MATE2-K, OCT3, OATs1–4 (OAT1, OAT2v1, OAT3, OAT4) and PEPT2.

## 2. Results

### 2.1. Vesicular Transport Inhibition (ABC) Assays

In the first step of our in vitro studies, we examined whether the [^177^Lu]Lu-PSMA-617 isotopologue, ([α,β-^3^H]Nal)Lu-PSMA-617, inhibited the transport of known positive control substrates. In mammalian HEK (human embryonic kidney) and insect Sf9 (*Spodoptera frugiperda)* cell lines stably expressing human BCRP, MDR1, MRP1 and MRP4 transporters, the possible inhibitory effects on BCRP-mediated estrone-3-sulfate (E3S), MDR1-mediated *N*-methyl quinidine (NMQ), MRP1-mediated estradiol-17-*β*-glucuronide (E_2_17βG) and MRP4-mediated dehydroepiandrosterone sulphate (DHEAS) transport at two different concentrations ([c] = 0.30, 3.00 µM) of the test substance, ([α,β-^3^H]Nal)Lu-PSMA-617, were investigated.

**Figure 4:**
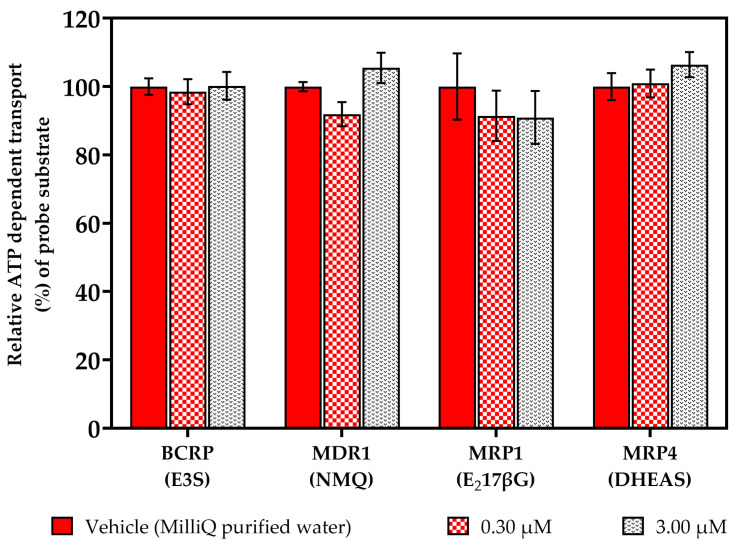
Vesicular transport inhibition assays in the presence of ([α,β-^3^H]Nal)Lu-PSMA-617 ([c] = 0.3, 3.0 µM). Inhibition studies of BCRP-mediated estrone-3-sulfate (E3S), MDR1-mediated *N*-methyl quinidine (NMQ), MRP1-mediated estradiol-17-*β*-glucuronide (E_2_17βG) and MRP4-mediated dehydroepiandrosterone sulphate (DHEAS) transport were undertaken. Data are expressed as mean (n = 3) ± SD (standard deviation). Values higher than 20% were defined as representing significant inhibition. Concentrations are nominal.

For all the substrates, no significant changes in the relative ATP-dependent transport values were observed in the presence of the test substance, ([α,β-^3^H]Nal)Lu-PSMA-617 (Figure 4). We considered inhibition values >20% as representing significant inhibition, which were never achieved in our studies. With the positive control inhibitors performing as expected, our data indicate that ([α,β-^3^H]Nal)Lu-PSMA-617 is no inhibitor of the tested ABC transporters. Detailed calculations and results are listed in SI2 (in Supplementary Materials).

### 2.2. Vesicular Transporter Substrate (SLC) Assays

Next, the potential nature of ([α,β-^3^H]Nal)Lu-PSMA-617 as an in vitro substrate of the human SLC transporters MATE1, MATE2-K, OCT3, OAT1-4 (OAT1, OAT2v1, OAT3, OAT4) and PEPT2 was assessed. 

**Figure 5:**
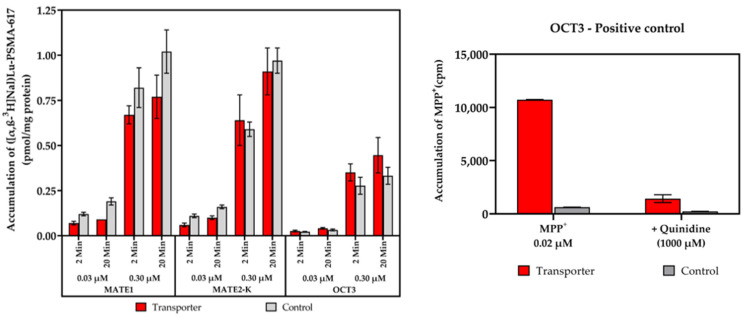
SLC transporter substrate assay of ([α,β-^3^H]Nal)Lu-PSMA-617 in MATE1-, MATE2-K- and OCT3-expressing and transporter-negative control cells at test substrate concentrations of [c] = 0.03/0.30 µM and incubation periods of t = 2/20 min. Positive control for OCT3 listed exemplary with MPP^+^ (1-Methyl-4-phenylpyridin-1-ium) as a probe substrate and quinidine as a corresponding inhibitor. Data are expressed as mean (n = 3) ± SD. Concentrations are nominal. Detailed calculations, conditions and reference inhibitors are listed in SI4.

At first, the accumulation of ([α,β-^3^H]Nal)Lu-PSMA-617 in the MATE1, MATE2-K and OCT3 transporters, stably expressed in the HEK and MDCKII cell lines, was examined at two different test substance concentrations ([c] = 0.03, 0.30 µM) and for two different incubation periods [t = 2, 20 min]. Figure 5 shows that no accumulation of the test substance in the MATE1, MATE2-K and OCT3 transporters took place. Comparing the obtained accumulation values in the transporter-expressing lines against the transporter-negative control cell lines, it is likely that ([α,β-^3^H]Nal)Lu-PSMA-617 is no in vitro substrate of the aforementioned transporters. Detailed results and calculations are listed in SI4 (in Supplementary Materials).

**Figure 6:**
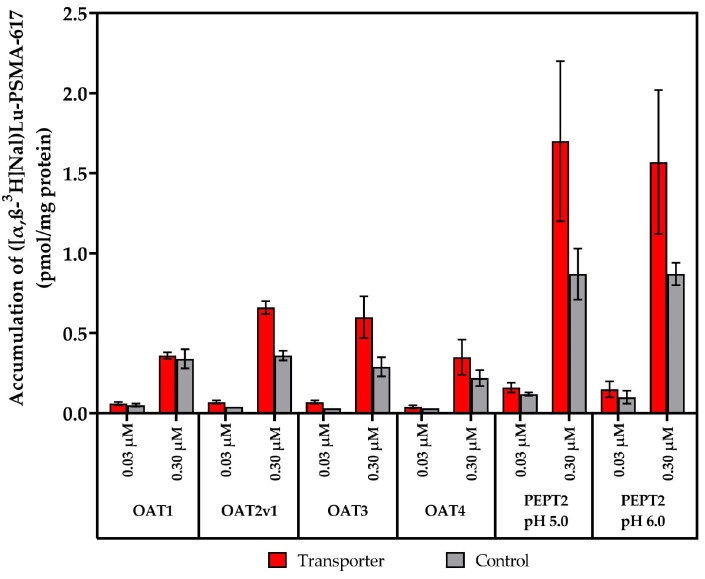
SLC transporter substrate assay of ([α,β-^3^H]Nal)Lu-PSMA-617 in OAT1, OAT2v1, OAT3, OAT4, PEPT2 (pH 5.0/6.0)-expressing and transporter-negative control cells at test substrate concentrations of [c] = 0.03, 0.30 µM and an incubation period of t = 20 min. Data are expressed as mean (n = 3) ± SD. Concentrations are nominal. Detailed calculations, conditions and reference inhibitors are listed in SI4.

Next, the nature of ([α,β-^3^H]Nal)Lu-PSMA-617 as an in vitro substrate was examined for the OAT1, OAT2v1, OAT3, OAT4 and PEPT2 transporters, stably expressed in the HEK and CHO (Chinese hamster ovarian) cell lines. As no accumulation of the test substance was observed for MATE1, MATE2K or OCT3, the next experiments were conducted at two different test substance concentrations ([c] = 0.03, 0.30 µM) and an incubation period of t = 20 min (Figure 6). In contrast to the results for the MATE1, MATE2-K and OCT3 transporters, higher accumulation values in the transporter-expressing cell lines were observed in comparison to in the applied controls.

To verify whether the observed accumulation of ([α,β-^3^H]Nal)Lu-PSMA-617 (Figure 6) in the cells resulted from uptake by OAT1, OAT2v1, OAT3, OAT4 or PEPT2, we repeated the previous SLC assays with the addition of the corresponding reference inhibitors. As inhibitors, probenecid (OAT1, OAT3), indomethacin (OAT2v1), benzbromarone (OAT4) and cefadroxil (PEPT2) were used, respectively.

**Figure 7:**
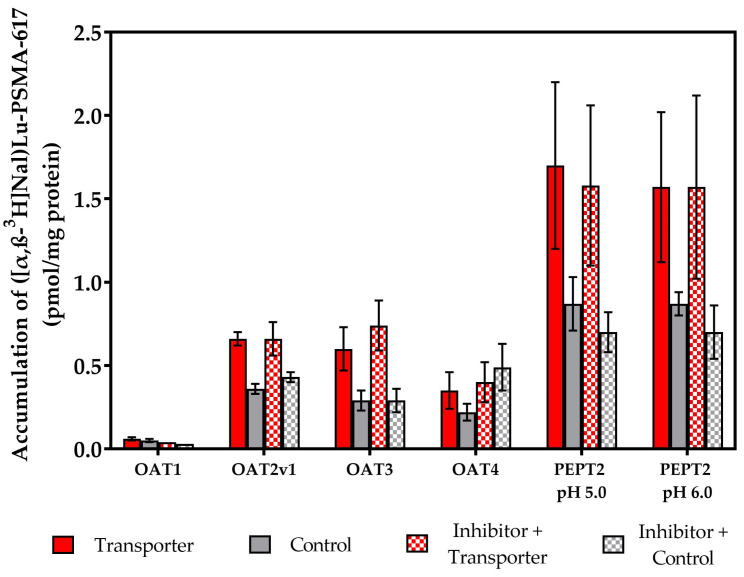
SLC transporter substrate assay of ([α,β-^3^H]Nal)Lu-PSMA-617 in OAT1, OAT2v1, OAT3, OAT4, PEPT2 (pH 5.0, 6.0)-expressing and transporter-negative control cells in both absence and presence of a corresponding reference inhibitor. Test substrate concentrations were [c] = 0.30 µM in all cases except for OAT1 ([c] = 0.03 µM) with incubation times of t = 20 min. Data are expressed as mean (n = 3) ± SD. Detailed conditions and inhibitors are listed in SI4.

In comparison to the uptake experiments displayed in Figure 6, the accumulation values showed no changes neither in the transporter-expressing nor the transporter-negative control cell lines when they were incubated with the corresponding reference inhibitors (Figure 7). This clearly indicates that ([α,β-^3^H]Nal)Lu-PSMA-617 is not taken up via the different tested SLC transporters. 

In summary, both our nominal and fold accumulation data (Table S9 in SI4 in Supplementary Materials) indicate no active accumulation of ([α,β-^3^H]Nal)Lu-PSMA-617 in any of the examined control and transporter-expressing cell lines. 

Hence, we conclude that ([α,β-^3^H]Nal)Lu-PSMA-617 is neither an inhibitor nor a substrate of the examined transporters. 

## 3. Discussion 

It is well reported that regardless of the increased PSMA expression on malignant PCa cells, severe accumulation and retention of [^177^Lu]Lu-PSMA-617 and [^225^Ac]Ac-PSMA-617 can occur in healthy kidneys and SGs despite them having a much lower PSMA expression [18,19,20]. Since they are inherently radiosensitive, this uptake critically limits the scope of PSMA-targeted radioligands’ application. Realizing this, a substantial effort has been made to improve the related side-effects and to decrease undesired SG uptake, e.g., through local cooling [37], injecting botulinum toxin A into affected areas [38] or co-administering cold PSMA ligands [15,39] and glutamate receptor binders, such as monosodium glutamate [40] or Tris-POC-2-PMPA [41], to minimize kidney retention. However, none of these measures have led to a significant reduction in side-effects such as xerostomia, and studies hint at unspecific uptake mechanisms for the respective PSMA moieties. Nonetheless, the majority of administered [^177^Lu]Lu-PSMA-617 (up to 70%) is reported to be quickly excreted via the renal route in urine within the first 24 h post injection [42]. To our knowledge, this is the first study to examine the role of ABC and SLC transporters, involved in many sensitive drug transport processes [23,33,43,44] and located in healthy SGs and kidneys, as regards undesired [^177^Lu]Lu-PSMA-617 and [^225^Ac]Ac-PSMA-617 uptake.

Focusing on the main excretory organs, the kidneys, could indicate the first mechanistic hints to elucidate the ADME processes for PSMA-617. In the kidneys, drug excretion is mediated through a vast array of transporters located in the apical and basolateral membranes of the renal proximal tubules [34]. In recent decades, multiple drug transporters have been explored and evaluated in their functions, becoming a central research item in drug pharmacokinetics [45]. Most recently, these aforementioned ABC and SLC transporters have also been detected in the SGs, albeit at different abundances [26]. 

Specifically, we investigated whether the toxic uptake of PSMA moieties in healthy SGs and kidneys might be mediated by the vital BCRP, MDR1, MRP1, MRP4 (ABC) and MATE1, MATE2-K, OCT3, OATs1-4 (OAT1, OAT2v1, OAT3, OAT4) and PEPT2 (SLC) transporters. Our results indicate that ([α,β-^3^H]Nal)Lu-PSMA-617, an isotopologue analog of [^177^Lu]Lu-PSMA-617, acts neither as an inhibitor nor a substrate of the ABC and SLC transporters potentially expressed in both the kidneys and SGs.

On a positive note, this lack of interaction can be seen as a major advantage for parallel multi-drug therapies being used alongside PSMA-based radionuclide therapies. In clinics, patients can be involved in multi-drug therapies, where unwanted drug–drug-interactions (DDIs) can occur and cause adverse effects or distort the desired treatment outcomes altogether, which might be especially pertinent in elderly patients [46]. As [^177^Lu]Lu-PSMA-617 acts as neither an inhibitor nor a substrate of the examined ABC and SLC transporters, the application of transporter-targeting drugs in multi-drug therapies should remain unhindered, and the probability of unwanted DDIs should be reduced. 

In contrast to PSMA expression [4,47], the abundance of transporters has been reported to decrease based on increasing age, lifestyle or the presence of additional diseases [34,48], accompanied by large interpersonal variability [26]. For instance, Wen et al. reported that a decline in the renal transporters—which was examined in cisplatin excretion studies—can be prevalent in older patients. They concluded that the excretion of cisplatin is significantly lower in patients of an advanced age (≥50 years), possibly due to a strong decrease in the abundance of MATE1, which, in turn, results in increased renal accumulation of cisplatin and subsequent nephrotoxicity [49]. In addition to this, Uddin et al. have reported that MATE1 transporters can show a severe sensitivity towards small-molecule inhibitors. In their study, 37 of 57 examined tyrosine kinase inhibitors (TKIs) potently inhibited MATE1 function in HEK298 cells by numbers > 80% through a non-competitive, reversible, substrate-independent mechanism, leading to a two-fold drop in renal oxaliplatin excretion [44]. As the overall uptake of [^177^Lu]Lu-PSMA-617 appears to be transporter-independent, both the abundance of vital transporters and its age-dependent decrease should not interfere with the delivery of PSMA-based radioligands to tumor cells, enabling a positive treatment outlook for all patients. While an interaction between PSMA-617 and the examined transporters is not present, we strongly believe its passive diffusion into the exquisitely radiosensitive [50] SGs and its affinities to other antigens/enzymes or PSMA homologues [51] to factor into its non-specific uptake, alongside structural features of PSMA-based radioligands such as their ionic charges and molecular sizes [19,21].

However, general uptake profiles can also correlate with patient-specific factors. In PCa patients of a younger age, inferior treatment responses and higher risks of biochemical recurrence have been observed [52]. This might be due to the PCa being at an early stage, as opposed to older patients being at advanced disease stages. Furthermore, a comparative study of black and white South African men revealed that higher incidences of PCa are prevalent in black men, potentially leading to a much higher uptake of PSMA-based radioligands [53]. Aside from age and ethnicity, overall health status might strongly impact undesired uptake through a weak immune system or limited renal function, possibly resulting from extensive pre-treatment regiments, co-morbidities or even natural causes. As a result, longer retention times for the applied radiopharmaceuticals can induce damage to inherently radiosensitive healthy organs due to unspecific uptake, premature radionuclide release and subsequent recoil effects.

Among many approaches to reducing toxic SG and kidney uptakes, the implementation of antibodies (>150 kDa) instead of small-molecule-based PSMA ligands (<1.5 kDa) is also known. As a matter of fact, PSMA was first identified with the monoclonal antibody (mAb) 7E11 [54,55], which led to the first generation of PSMA-targeting agents, such as the ^111^In-labeled 7E11-C5.3 (ProstaScint^®^), which is conjugated to a GYK-DTPA chelator consisting of the tripeptide glycine (G), L-tyrosine (Y) and L-lysine (K) and pentetic acid (DTPA) [56,57]. While successful at targeting metastatic lesions, 7E11-C5.3 binds only to an intracellular epitope of PSMA, critically limiting its use in binding viable tumor cells [20]. Subsequently, new mAbs have been developed to target the extracellular domain, which make up to 95% of PSMA itself [58,59]. Liu et al. developed J591 in 1997, a de-immunized mAb with a 1 nM binding affinity to PSMA_ext_ [60,61], which has been successfully radiolabeled with ^89^Zr [62], ^90^Y [63], ^111^In [64], ^131^I [61], ^177^Lu [65], ^213^Bi [66] and ^225^Ac [67,68,69], and contrary to PSMA-617, shows a much lower distribution in the SGs and kidneys [68]. Still, limitations remain, as antibodies exhibit slow tumor penetration characteristics and much longer plasma circulation times, the latter potentially leading to dose-limiting hemato- or myelotoxicities being prevalent even in third-generation mAbs [70].

Bioengineering nanobodies has aided in this matter by reducing the plasma circulation times and enabling faster tumor penetration. In vitro displacement studies of the PSMA-targeted nanobody [^177^Lu]Lu-JVZ-007 suggest a binding behavior similar to that of the mAb J591. Here, [^177^Lu]Lu-JVZ-007 was not displaced by increased concentrations of unlabeled PSMA-617 and PSMA I&T and vice versa, strongly suggesting an alternate PSMA binding site for this nanobody [71]. 

Lucaroni et al. hypothesized the cross-reactivity with glutamate carboxypeptidase III (GCPIII), a homologue of GCPII (PSMA), to be responsible for the undesired SG and kidney uptake of PSMA-targeted ligands [72], stirring up heated debate. Lee et al. contradicted this hypothesis by injecting [^68^Ga]Ga-PSMA-11 into PSMA-null mice and observing a greatly reduced uptake by healthy kidneys and SGs [73], asserting GCPII selectivity over GCPIII selectivity. 

The hypothesis on GCPII selectivity was further supported by Huang et al. through the implementation of negatively charged side-chain linkers into the PSMA backbone to yield [^68^Ga]Ga-JB-1498. In their biodistribution study, [^68^Ga]Ga-JB-1498 demonstrated a significantly decreased kidney and SG uptake in PSMA wild-type mice in comparison to [^68^Ga]Ga-PSMA-11. If unwanted kidney and SG uptake could be due to selective GCPII binding, the implementation of ionically charged linkers could circumvent this effect according to a transport mechanism yet to be identified [74].

While the molecular sizes, ionic charges and potential binding sites of PSMA-targeted ligands might play a role in understanding its possible transport mechanism, we conclude that they are not relevant to the ABC and SLC transporters examined in our study.

## 4. Materials and Methods

### 4.1. Chemicals, Reagents and Instruments

All the reagents and solvents were of analytical grade and were purchased unless noted otherwise. The purified water used herein was prepared using a Millipore Milli-Q Reference system. 

The adenosine 5′-monophosphate sodium salt (AMP), adenosine 5′-triphosphate disodium salt hydrate (ATP), benzbromarone, cefadroxil, dehydroepiandrosterone sulfate (DHEAS), β-estradiol 17-(β-D-glucuronide) sodium salt (E_2_17βG), estrone-3-sulfate sodium salt (E3S), glycylsarcosine (Gly-Sar), guanosine 3′,5′-cyclic monophosphate (cGMP), indomethacin, Ko143 hydrate, MES hydrate (2-(*N*-morpholino)ethanesulfonic acid), 1,1-dimethylbiguanide hydrochloride (Metformin), MK-571, probenecid, pyrimethamine and Valspodar were purchased from Sigma-Aldrich (St Louis, MO, USA). The ^3^H-DHEAS ([1,2,6,7- ^3^H(*N*)]-dehydroepiandrosterone sulfate sodium salt) and estradiol 17β-D-glucuronide [Estradiol-6,7-^3^H(*N*)] were purchased from PerkinElmer (Waltham, MA, USA). The [^3^H]Estrone sulfate was purchased from Radiolab (Szeged, Hungary). The [^3^H]glycylsarcosine, [8-^3^H]-guanosine 3’,5’-cyclic phosphate ammonium salt, metformin hydrochloride, [biguanidine-^14^C] and [adenine-2,8-^3^H]tenofovir were purchased from Moravek Biochemicals (Brea, CA, USA). The [^3^H]-*N*-methyl quinidine and *N*-methyl quinidine (NMQ) were supplied by SOLVO Biotechnology (Szeged, Hungary). The tenofovir was purchased from Sequoia Research Products Ltd. (Pangbourne, UK).

Kinetic solubility assessments were verified using simple optical microscopy evaluation (50 times magnification). The inhibition and transport of the radiolabeled substances were followed using radio-detection instruments, including a PerkinElmer (Waltham, MA, USA) MicroBeta2 liquid scintillation counter (LSC) and a BMG Labtech (Offenburg, Germany) FLUOstar Omega multifunctional microplate reader. 

### 4.2. Cell Culture

The transporter assays were performed using cell lines stably transfected with human efflux (ABC) or uptake (SLC) transporters. The BCRP, MDR1 and MRP4 efflux transporters, as well as the OAT1, OAT2v1, OAT3, OAT4 and OCT3 uptake transporters, were expressed in mammalian human embryonic kidney cells (HEK293); the MRP1 transporter was expressed in the pupa ovarian tissue of the fall armyworm (*Spodoptera frugiperda* [Sf9]); the MATE1 and MATE2-K uptake transporters were expressed in a mammalian subclone of Madin–Darby canine kidney cells (MDCKII) and the PEPT2 uptake transporter was expressed in a mammalian sub-clone of adult female Chinese hamster ovarian cells (CHO-K1). The control cell lines included MDCKII-CAT-Fin, mock-transfected HEK293 and CHO-K1. The cell lines were cultured at 37 °C in a humidified atmosphere containing 5% CO_2_. All the cell lines except for CHO-K1 and its derivatives were cultured in Dulbecco’s Modified Eagle Medium (DMEM) supplemented with 4.5 g/L glucose. CHO-K1-PEPT2 and the control CHO-K1 were cultured in DMEM-12. The cell lines were provided, cultured and subsequently analyzed in in vitro assays under non-GLP conditions by SOLVO Biotechnology, a Charles River company (Szeged, Hungary).

### 4.3. Synthesis and Test Substance Sample Preparation

PSMA-617 was synthesized according to the previously reported procedures (chemical purity > 97%) [7]. The novel tritium- and cold lutetium-labeled ([α,β-^3^H]Nal)Lu-PSMA-617 (chemical purity > 99%, radiochemical purity > 99%) was prepared according to our recently published work [36] and was kept in an EtOH:H_2_O 1:1 solution below −20 °C. The total activity of ([α,β-^3^H]Nal)Lu-PSMA-617 was 37 MBq (1 mCi), with a molar activity of 876.9 GBq/mmol (23.7 Ci/mmol). The concentration was 37.97 µM, and the radioactivity per well in the transporter assays was 7.9 kBq (0.2133 µCi).

A 2 mM stock solution and a dilution series of ([α,β-^3^H]Nal)Lu-PSMA-617 ([c] = 1.0, 0.30 and 0.03 mM) were prepared in MilliQ purified water and utilized in the conducted assays (100-fold dilution in the vesicular transport inhibition assays, 1000-fold dilution in the SLC transporter substrate assays). The concentration of organic solvent in the assay buffers did not exceed 1.5% (*v*/*v*) in the vesicular transport and 1.0% (*v*/*v*) in the substrate uptake assays. 

### 4.4. Experimental Methods for the Vesicular Transport Inhibition Assays (ABC Transporter)

The vesicular transport inhibition assays were conducted at two different test substance concentrations ([c] = 0.30, 3.00 µM) incubated with membrane vesicle preparations (total protein content: 50 μg/well—MDR1, MRP1, MRP4; 12.5 μg/well—BCRP). The used positive control substrates and corresponding reference inhibitors with their respective concentrations are listed in Table S2 in SI2.

The incubations were carried out in the presence of 4 mM ATP or AMP to distinguish between transporter-mediated uptake and passive diffusion into the vesicles. ([α,β-^3^H]Nal)Lu-PSMA-617 ([c] = 0.03, 0.30 µM) was added to 50 μL of the corresponding probe-substrate-containing assay solution in 0.75 μL of solvent (1% of the final incubation volume), as described in Table S2 in SI2. Afterwards, the reaction mixtures were pre-incubated for 15 min at 37 °C for MRP1 or at 32 °C for BCRP, MDR1 and MRP4, respectively. The transporter assay was initiated through the addition of 25 μL of pre-warmed 12 mM MgATP (or 12 mM AMP in assay buffer as a background control). The reactions were quenched through the addition of 200 μL of ice-cold washing buffer and immediate filtration via glass fiber filters mounted onto a 96-well plate (filter plate). The filters were washed (5 × 200 μL of ice-cold washing buffer) and dried, and the amount of probe substrate inside the filtered vesicles was determined using LSC. The experiments were conducted in triplicate. Detailed results and calculations are listed in SI2, and the treatment groups and detailed controls are listed in SI3 (in Supplementary Materials).

### 4.5. Experimental Methods for the Transporter Substrate Assays (SLC Transporter)

The described cells were plated onto standard 24-well tissue culture plates at densities of 5 × 10^5^ cells/well. The uptake was investigated at two different test substance concentrations ([c] = 0.03, 0.30 µM) of ([α,β-^3^H]Nal)Lu-PSMA-617 using cells overexpressing the respective uptake transporter and control cells. The OAT1, OAT2v1, OAT3, OAT4 and PEPT2 uptake experiments were carried out in the absence and presence of a corresponding reference inhibitor to determine whether or not ([α,β-^3^H]Nal)Lu-PSMA-617 was actively taken up into the cells. Before starting the experiment, the cells were prepared by removing the medium and washing the cells twice with 300 µL of assay buffer. The cellular ([α,β-^3^H]Nal)Lu-PSMA-617 uptake into the cells was measured according to the addition of 300 µL assay buffer containing ([α,β-^3^H]Nal)Lu-PSMA-617 and their incubation at 37 °C. The reactions were quenched by removing the buffer containing ([α,β-^3^H]Nal)Lu-PSMA-617, and the cells were washed twice with 300 µL of assay buffer. The cells were lysed by adding 300 µL of 0.1N NaOH, they were incubated for 10 min at 37 °C and 100 µL samples were taken from all the wells.

The amount of PSMA-617 in the cell lysate was determined using LSC. The amount of protein in each well was quantified using the BCA kit for protein determination (Sigma-Aldrich, St Louis, MO, USA). Positive controls were performed using a separate 96-well plate format according to Table S8 in SI4. Detailed results and calculations are listed in SI4, and the treatment groups and detailed controls are given in SI5.

## 5. Conclusions

In this study, using the novel isotopologue ([α,β-^3^H]Nal)Lu-PSMA-617, we examined the possible interaction of PSMA-617 with ABC (BCRP, MDR1, MRP1, MRP4) and SLC transporters (MATE1, MATE2-K, OCT3, OAT1, OAT2v1, OAT3, OAT4, PEPT2). Our results do not indicate ([α,β-^3^H]Nal)Lu-PSMA-617 is an inhibitor or substrate of any of the aforementioned transporters. Taking these data into consideration, the mechanism of non-PSMA-specific [^177^Lu]Lu-PSMA-617 and [^225^Ac]Ac-PSMA-617 uptake into the salivary glands and kidneys still remains elusive and needs to be further investigated.

## Data Availability

Upon request, the presented data can be made available from the corresponding author.

## Supplementary Materials

The following supporting information can be downloaded at https://www.mdpi.com/article/10.3390/ph17040513/s1. SI1: Solubility assessment of PSMA-617, SI2: Results of vesicular transport inhibition assays (ABC), SI3: Treatment groups and controls of vesicular transport inhibition assays (ABC), SI4: Results of transporter substrate assays (SLC), SI5: Treatment groups and controls of transporter substrate assays (SLC).
