# Supplementary material for: Human ABC and SLC Transporters: The Culprit Responsible for Unspecific PSMA-617 Uptake?
Source: Pharmaceuticals (Basel). 2024 Apr 16;17(4):513. doi: 10.3390/ph17040513 (PMC11053447; doi:10.3390/ph17040513)
Supplement: Supplementary file 1 [file pharmaceuticals-17-00513-s001.zip › pharmaceuticals-2929931-supplementary.pdf]

Original Article

Supplementary Info

Human ABC and SLC transporters: the culprit responsible for unspecific PSMA-617 uptake?

Harun Taş[1], Gábor Bakos[1], Ulrike Bauder-Wüst[1], Martin Schäfer[2], Yvonne Remde[2], Mareike Roscher[2], Martina Benešová-Schäfer[1,*]

Contents

SI1. Solubility assessment of PSMA-617 2

SI2. Results of vesicular transport inhibition assays (ABC) 3

Calculation of ATP-dependent transport and inhibition for vesicular transport inhibition 3

2.1 Breast cancer resistance protein BCRP 5

2.2 Multidrug resistance protein MDR1 6

2.3 Multidrug resistance-related protein MRP1 7

2.4 Multidrug resistance-related protein MRP4 8

SI3. Treatment groups & controls of vesicular transport inhibition assays (ABC) 9

SI4. Results of transporter substrate assays (SLC) 10

Calculation of fold accumulation values for SLC transporter substrate assays 11

4.1 Multidrug and toxin extrusion protein MATE1 12

4.2 Multidrug and toxin extrusion protein MATE2-K 13

4.3 Organic anion transporter OAT1 14

4.4 Organic anion transporter OAT2v1 15

4.5 Organic anion transporter OAT3 16

4.6 Organic anion transporter OAT4 17

4.7 Organic cation transporter OCT3 18

4.8 Peptide transporter PEPT2 19

SI5. Treatment groups & controls of transporter substrate assays (SLC) 21

SI1. Solubility assessment of PSMA-617

The kinetic solubility of unlabeled PSMA-617 was assessed to ensure proper *in vitro* conditions. For this purpose, a five-step dilution series of said compound was prepared in corresponding transporter assay buffers listed in detail in **Table S1**.

A stock solution of unlabeled PSMA-617 in MilliQ purified water was diluted to samples of five different concentrations of [c] = 0.375, 0.75, 1.5, 1.888, 3.0 μM for vesicular transport inhibition assays (ABC) and of [c] = 0.063, 0.125, 0.25, 0.5, 1.0 μM for transporter substrate assays (SLC). Subsequently, the samples were mixed with the appropriate transporter assay buffers. In case of vesicular transport inhibition assays (ABC), 96-wells plates and a 100-fold dilution factor were applied, whereas for SLC transporter substrate assays, 15 mL tubes and a 1000-fold dilution factor were applied. Afterwards, the solutions were incubated for 15 minutes at 37°C, or at 32°C in case of buffers for testing BCRP, MDR1 and MRP4, respectively. The incubated solutions were then evaluated by optical microscopy (50 times magnification) to determine whether any visible crystals or particles formed. The experiment was performed in triplicate.

Table S1: Experimental conditions of solubility assays.

Assay type	Assay buffer (pH)	Human transporter	Incubation conditions	Dilution factor	Final solubility concentrations [μM]
Vesicular transport inhibition assay (ABC)	Transport buffer with sucrose (7.4)	BCRP, MDR1, MRP4	32°C, 15 min	100	0.375, 0.750, 1.500, 1.888 and 3.000
	Transport buffer without sucrose (7.4)	MRP1	37°C, 15 min		
Transporter substrate assay (SLC)	KH (7.4)	OCT3	37°C, 15 min	1000	0.063, 0.125, 0.250, 0.500 and 1.000
	KH (8.0)	MATE1, MATE2-K			
	HBSS (7.4)	OAT1, OAT2v1, OAT3, OAT4			
	KH with MES (5.0)	PEPT2			
	KH with MES (6.0)				

KH: Krebs-Henseleit buffer, HBSS: Hank's balanced salt solution, MES: 2-(*N*-morpholino) ethanesulfonic acid.

SI2. Results of vesicular transport inhibition assays (ABC)

Calculation of ATP-dependent transport and inhibition for vesicular transport inhibition

For all wells the amount of the translocated probe substrate was determined in counts per minute (cpm). ATP-dependent transport (pmol/mg protein/min) is calculated for each concentration using the following formula:

$$ATP - dependent\ accumulation = \left(\frac{\frac{Acc_{ATP}}{T_{CPM}} \cdot V \cdot CC_{sub}}{\frac{Prot}{1000}/t}\right) - \left(\frac{\frac{Acc_{AMP}}{T_{CPM}} \cdot V \cdot CC_{sub}}{\frac{Prot}{1000}/t}\right)$$

with:

- Acc_{ATP}: probe substrate accumulation with ATP (cpm)
- Acc_{AMP}: probe substrate accumulation with AMP (cpm)
- T_{cpm}: cpm in dosing solution V: volume per well (µL)
- CC_{sub}: probe substrate concentration (µM)
- Prot: total protein per well (mg)
- t: incubation time (min)

Relative ATP-dependent transport (%) values were calculated using the following equation:

$$Relative\ ATP - dependent\ transport\ (\%) = \frac{A - B}{C - D} \cdot 100$$

with:

- A: amount of translocated substrate in the presence of test substance (TS) and ATP
- B: amount of translocated substrate in the presence of test substance (TS) and AMP
- C: amount of translocated substrate in the presence of solvent and ATP
- D: amount of translocated substrate in the presence of solvent and AMP

Relative inhibition (%) is calculated by setting probe substrate transport in the absence of test substance as 100%. Inhibition values higher than 20% were defined as a significant inhibition.

$$Relative\ inhibition\ (\%) = \frac{Acc_{Specific,x}}{Acc_{Specific,vehicle}} \cdot 100$$

with:

- $Acc_{specific,x}$: transporter specific accumulation for a given sample (pmol/mg/min)
- $Acc_{specific,vehicle}$: transporter specific accumulation for a solvent control (pmol/mg/min)

Table S2: Experimental conditions for test systems and vesicular transport inhibition assays.

Human Transporter (Transfected Cell Line)	Protein content per well [μg]	Incubation conditions	Positive control substrate / Reference inhibitor [μM]
BCRP (HEK293)	12.5	32°C, 1 min	E3S (1.0) / Ko143 (0.2)
MDR1 (HEK293)	50	32°C, 1 min	NMQ (1.0) / Valspodar (1.0)
MRP1 (Sf9)	50	37°C, 3 min	$E_2$17βG (0.2) / Benzbromarone (200)
MRP4 (HEK293)	50	32°C, 1.5 min	DHEAS (0.5) / MK-571 (150)

E3S: Estrone-3-sulfate, NMQ: *N*-methyl quinidine, $E_2$17βG: Estradiol-17-β-glucuronide, DHEAS: Dehydroepiandrosterone sulfate.

2.1 Breast cancer resistance protein BCRP

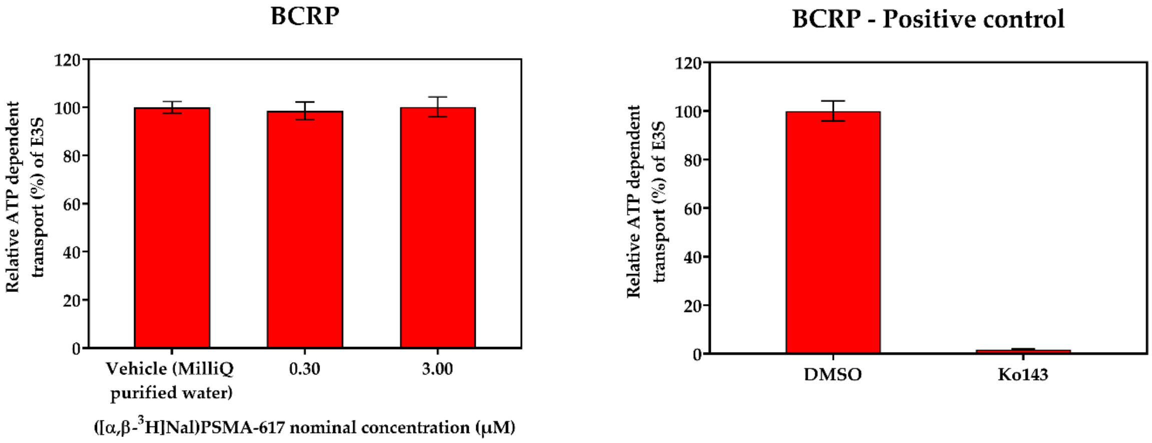

Figure S1: Graphical representation of the inhibition of BCRP-mediated estrone-3-sulfate (E3S) transport by ([α,β-^{3}H]Nal)Lu-PSMA-617 in a two-concentration vesicular transport inhibition assay. Data are expressed as mean (n=3) ± SD.

Table S3: Inhibition induced by ([α,β-^{3}H]Nal)Lu-PSMA-617 on the BCRP-mediated transport of estrone-3-sulfate (E3S) measured in the vesicular transport inhibition assay.

Compound	Nominal concentration [μM]	ATP dependent transport [pmol/mg/min]	Relative ATP dependent transport [% of control]	Relative inhibition [%]
([α,β-^{3}H]Nal) Lu-PSMA-617	3.00	23687.00 ± 869.33	100.22 ± 4.06	0
	0.30	23285.00 ± 769.03	98.5 ± 3.67	1
Test substance solvent (MilliQ purified water)	< 1.5 % (*v*/*v*)	23636.00 ± 405.14	100.00 ± 2.42	0
**Positive control**				
Ko143	0.20	374.67 ± 58.76	1.78 ± 0.28	98
Probe substrate solvent (DMSO)	< 1.5 % (*v*/*v*)	21100.67 ± 613.16	100.00 ± 4.11	0

Data are expressed as mean (n=3) ± SD.

2.2 Multidrug resistance protein MDR1

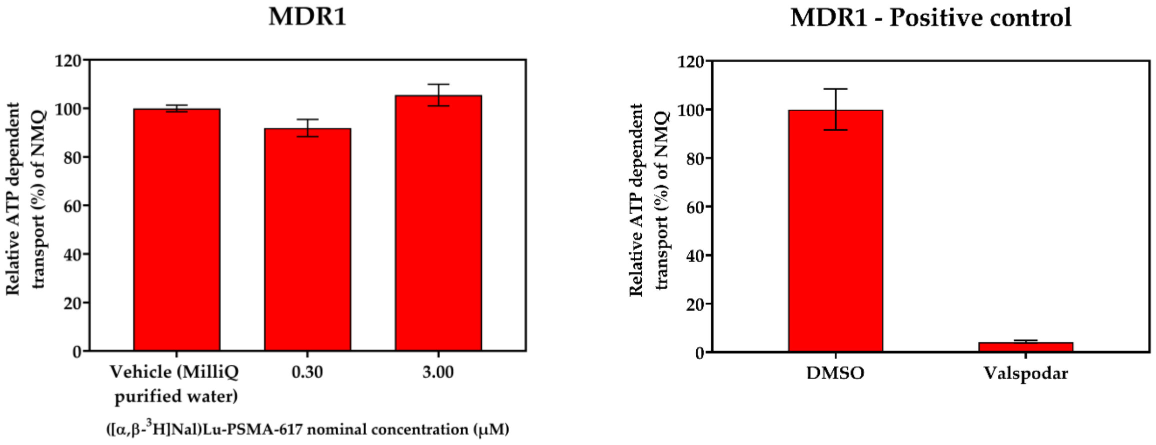

Figure S2: Graphical representation of the inhibition of MDR1-mediated *N*-methyl quinidine (NMQ) transport by ([α,β-^{3}H]Nal)Lu-PSMA-617 in two-concentration vesicular transport inhibition assay. Data are expressed as mean (n=3) ± SD.

Table S4: Inhibition induced by ([α,β-^{3}H]Nal)Lu-PSMA-617 on the MDR1-mediated transport of *N*-methyl quinidine (NMQ) measured in the vesicular transport inhibition assay.

Compound	Nominal concentration [µM]	ATP dependent transport [pmol/mg/min]	Relative ATP dependent transport [% of control]	Relative inhibition [%]
([α,β-^{3}H]Nal) Lu-PSMA-617	3.00	5625.67 ± 230.64	105.48 ± 4.44	-5
	0.30	4902.67 ± 183.91	91.93 ± 3.56	8
Test substance solvent (MilliQ purified water)	< 1.5 % (*v*/*v*)	5333.33 ± 51.29	100.00 ± 1.36	0
**Positive control**				
Valspodar	1.00	214.00 ± 28.47	4.28 ± 0.62	96
Probe substrate solvent (DMSO)	< 1.5 % (*v*/*v*)	5000.67 ± 298.23	100.00 ± 8.43	0

Data are expressed as mean (n=3) ± SD.

2.3 Multidrug resistance-related protein MRP1

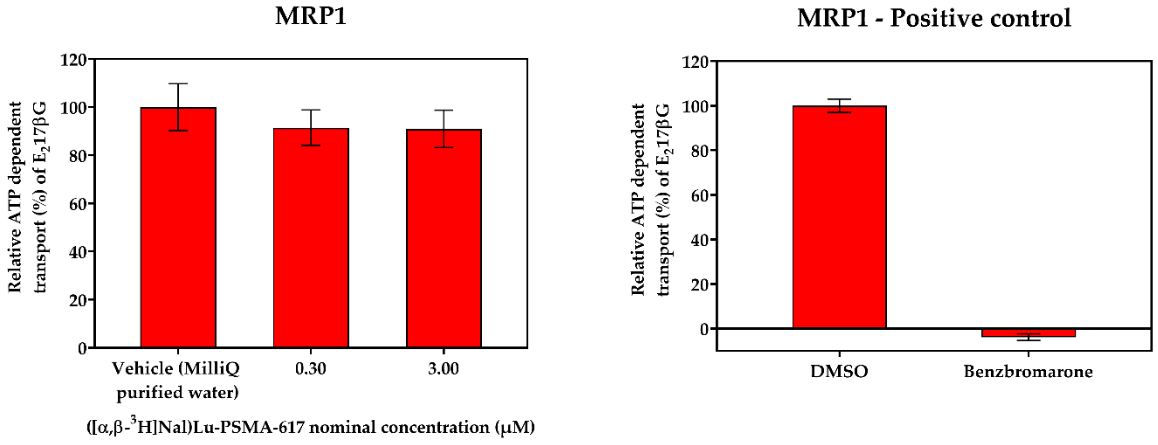

Figure S3: Graphical representation of inhibition of MRP1-mediated estradiol-17-β-glucuronide ($E_217\beta G$) transport by ([α,β-^{3}H]Nal)Lu-PSMA-617 in two-concentration vesicular transport inhibition assay. Data are expressed as mean (n=3) ± SD.

Table S5: Inhibition induced by ([α,β-^{3}H]Nal)Lu-PSMA-617 on the MRP1-mediated transport of estradiol-17-β-glucuronide ($E_217\beta G$) measured in the vesicular transport inhibition assay.

Compound	Nominal concentration [µM]	ATP dependent transport [pmol/mg/min]	Relative ATP dependent transport [% of control]	Relative inhibition [%]
([α,β-^{3}H]Nal) Lu-PSMA-617	3.00	730.00 ± 37.02	90.95 ± 7.76	9
	0.30	734.00 ± 31.29	91.45 ± 7.39	9
Test substance solvent (MilliQ purified water)	< 1.5 % (*v*/*v*)	802.67 ± 55.11	100.00 ± 9.71	0
**Positive control**				
Benzbromarone	200.0	-26.33 ± 10.19	-3.87 ± 1.50	104
Probe substrate solvent (DMSO)	< 1.5 % (*v*/*v*)	680.00 ± 14.28	100.00 ± 2.97	0

Data are expressed as mean (n=3) ± SD.

2.4 Multidrug resistance-related protein MRP4

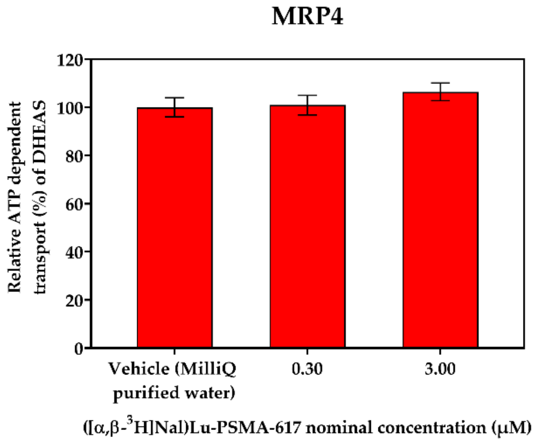

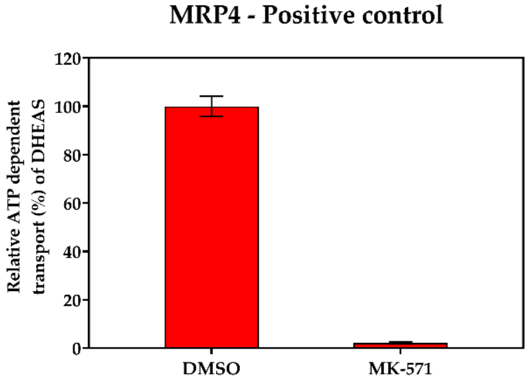

Figure S4: Graphical representation of the inhibition of MRP4-mediated dehydroepiandrosterone-sulphate (DHEAS) transport by ([α,β-^{3}H]Nal)Lu-PSMA-617 in two-concentration vesicular transport inhibition assay. Data are expressed as mean (n=3) ± SD.

Table S6: Inhibition induced by ([α,β-^{3}H]Nal)Lu-PSMA-617 on the MRP4-mediated transport of dehydroepiandrosterone-sulphate (DHEAS) measured in the vesicular transport inhibition assay.

Compound	Nominal concentration [μM]	ATP dependent transport [pmol/mg/min]	Relative ATP dependent transport [% of control]	Relative inhibition [%]
([α,β-^{3}H]Nal) Lu-PSMA-617	3.00	14510.33 ± 290.35	106.44 ± 3.66	-6
	0.30	13761.00 ± 400.40	100.94 ± 4.07	-1
Test substance solvent (MilliQ purified water)	< 1.5 % (*v*/*v*)	13632.67 ± 380.46	100.00 ± 3.95	0
**Positive control**				
MK-571	150.0	256.00 ± 59.15	2.06 ± 0.48	98
Probe substrate solvent (DMSO)	< 1.5 % (*v*/*v*)	12404.00 ± 367.68	100.00 ± 4.19	0

Data are expressed as mean (n=3) ± SD.

SI3. Treatment groups & controls of vesicular transport inhibition assays (ABC)

Treatment groups applied in the vesicular transport inhibition assays are listed in the table below:

Table S7: Treatment groups of vesicular transport inhibition assays (ABC).

Treatment groups in 96-well plate format	No. of wells
PSMA-617 (0.30 and 3.00 µM, nominal) in the respective transport buffer with ATP	3 per TS concentration
PSMA-617 (0.30 and 3.00 µM, nominal) in the respective transport buffer with AMP	
TA solvent (MilliQ purified water) control with ATP	3
TA solvent (MilliQ purified water) control with AMP	3
Reference inhibitor solvent (DMSO) control with ATP	3
Reference inhibitor solvent (DMSO) control with AMP	3
Reference inhibitor in the respective transport buffer with ATP	3
Reference inhibitor in the respective transport buffer with AMP	3

TS: Test substance. For the respective transport buffer see **S1 - Table S1**.

For controls:

- Incubation with AMP provided background activity values for all data points.
- Incubation with probe substrate (solvent control) provided 100% activity values.
- A reference inhibitor served as a positive control for inhibition.

SI4. Results of transporter substrate assays (SLC)

Table S8: Parameters of positive controls, control cell lines and plating for SLC transporters assays.

Human Transporter (Cell line)	Incubation conditions at 37°C [min]	Positive control substrate/ Reference inhibitor [μM]	Control cell line	Special treatment	Incubation prior to assay [h]	Assay buffer (pH)
MATE1 (MDCKII-MATE1-Fin)	15	Metformin (10.0) / Pyrimethamine (1.0)	MDCKII-CAT-Fin	-	24	KH (8.0)
MATE2-K (MDCKII-MATE2K-Fin)	5	Metformin (10.0) / Pyrimethamine (10.0)	MDCKII-CAT-Fin	-	24	KH (8.0)
OAT1 (HEK293-OAT1-LV)	2	Tenofovir (5.0) / Probenecid (300)	Mock-B-HEK293	Poly-D-lysine coated plate	16-24	HBSS (7.4)
OAT2v1 (HEK293-OAT2v1-LV)	1	cGMP (1.5) / Indomethacin (300)	Mock-B-HEK293	Poly-D-lysine coated plate	16-24	HBSS (7.4)
OAT3 (HEK293-OAT3-LV)	1	E3S (1.0) / Probenecid (500)	Mock-B-HEK293	Poly-D-lysine coated plate	16-24	HBSS (7.4)
OAT4 (HEK293-OAT4-LV)	1	E3S (1.0) / Benzbromarone (200)	Mock-B-HEK293	Poly-D-lysine coated plate	16-24	HBSS (7.4)
PEPT2 (CHO-K1-PEPT2)	8	Gly-Sar (4.0) / Cefadroxil (200)	CHO-K1	-	24	KH-MES (5.0)
PEPT2 (CHO-K1-PEPT2)	8	Gly-Sar (4.0) / Cefadroxil (200)	CHO-K1	-	24	*KH-MES (6.0)
OCT3 (HEK293-OCT3-LV)	3	MPP^+ (0.02) / Quinidine (1000)	Mock-HEK293	Poly-D-lysine coated plate	16-24	KH (7.4)

KH: Krebs-Henseleit buffer, HBSS: Hank's balanced salt solution, MES: 2-(*N*-morpholino) ethanesulfonic acid, cGMP: Guanosine cyclic phosphate, E3S: Estrone-3-sulfate, Gly-Sar: Glycylsarcosine, MPP^+: 1-methyl-4-phenylpyridinium.

Calculation of fold accumulation values for SLC transporter substrate assays

The fold accumulation value was defined as the ratio of uptake of the test substance (TS) or probe substrate into transfected and control cells. If the fold accumulation is > 2 and can be inhibited by a known inhibitor of the transporter, the test substance can be considered as a substrate of the respective transporter.

$$Fold\ accumulation = \frac{\mathrm{UPT}_{TRP}}{\mathrm{UPT}_{CTRL}}$$

with:

- $\mathrm{UPT}_{\mathrm{TRP}}$: accumulated amount of test substance (TS) or probe substrate in transfected cells normalized by protein content [pmol/mg protein]
- $\mathrm{UPT}_{\mathrm{CTRL}}$: accumulated amount of test substance (TS) or probe substrate in control cells normalized by protein content [pmol/mg protein]

Table S9: Maximum transporter-specific fold accumulation calculations and substrate assessment of ([α,β-^{3}H]Nal)Lu-PSMA-617 in SLC transporters.

Human transporter	Max. transporter-specific fold accumulation		Reference inhibitor	Incubation time [min]	Test substance concentration [µM]
	Without inhibitor	With inhibitor			
MATE1	0.81	NA	-	2	0.3
MATE2-K	1.08	NA	-	2	0.3
OCT3	1.34	NA	-	20	0.3
OAT1	1.17	1.10	Probenecid	20	0.03
OAT2v1	1.83	1.55	Indomethacin	20	0.3
OAT3	2.09	2.52	Probenecid	20	0.3
OAT4	1.58	0.81	Benzbromarone	20	0.3
PEPT2 (pH 5.0)	1.95	2.27	Cefadroxil	20	0.3
PEPT2 (pH 6.0)	1.81	2.26		20	0.3

NA: not analyzed. Test substances were considered as substrates in case of fold accumulation values > 2 combined with inhibition values higher than 50%.

4.1 Multidrug and toxin extrusion protein MATE1

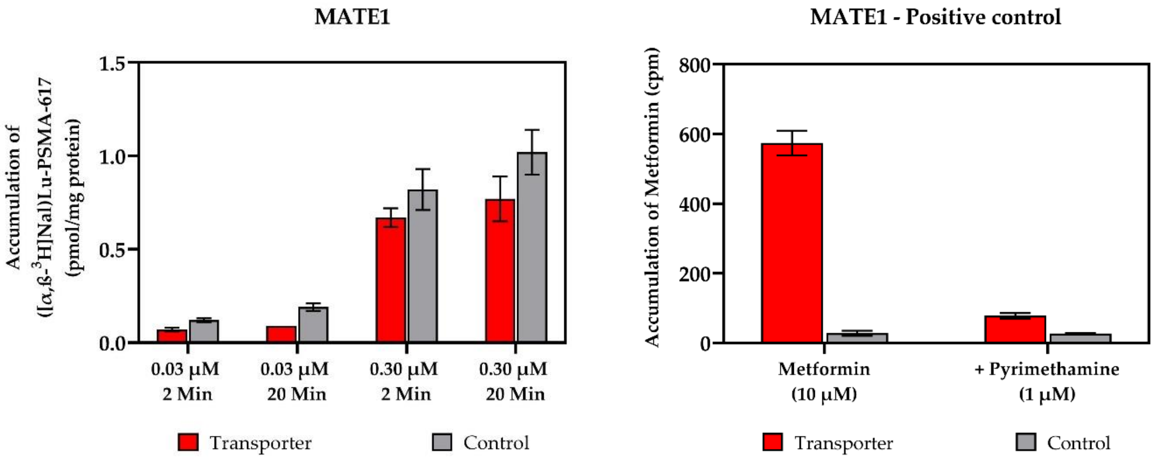

Figure S5: Graphical representation of ([α,β-^{3}H]Nal)Lu-PSMA-617-accumulation in MATE1 transporter expressing (MDCKII-MATE1-Fin) and control cells (MDCKII-CAT-Fin) applying 2- and 20-minute incubation periods, measured in SLC transporter substrate assay. Data are expressed as mean (n=3) ± SD. Concentrations are nominal.

Table S10: ([α,β-^{3}H]Nal)Lu-PSMA-617-accumulation in MATE1 transporter-expressing (MDCKII-MATE1-Fin) and control cells (MDCKII-CAT-Fin) applying 2- and 20-minute incubation periods, measured in SLC transporter substrate assay.

Compound	Conditions		Accumulation (pmol/mg protein)			Fold accumulation
	Nominal concentration [µM]	Minutes	In MATE1 transporter expressing cells	In control cells	Active transport	
([α,β-^{3}H]Nal) Lu-PSMA-617	0.03	2	0.07 ± 0.01	0.12 ± 0.01	-0.05 ± 0.02	0.60
		20	0.09 ± 0.00	0.19 ± 0.02	-0.10 ± 0.02	0.49
	0.30	2	0.67 ± 0.05	0.82 ± 0.11	-0.15 ± 0.12	0.81
		20	0.77 ± 0.12	1.02 ± 0.12	-0.26 ± 0.17	0.75
**Positive control (accumulation in cpm)**						
Metformin	10	15	574.00 ± 35.37	27.67 ± 7.37	546.33 ± 36.13	20.75
+ Pyrimethamine	+ 1		77.67 ± 7.57	27.00 ± 2.00	50.67 ± 7.83	2.88

Data are expressed as mean (n=3) ± SD.

4.2 Multidrug and toxin extrusion protein MATE2-K

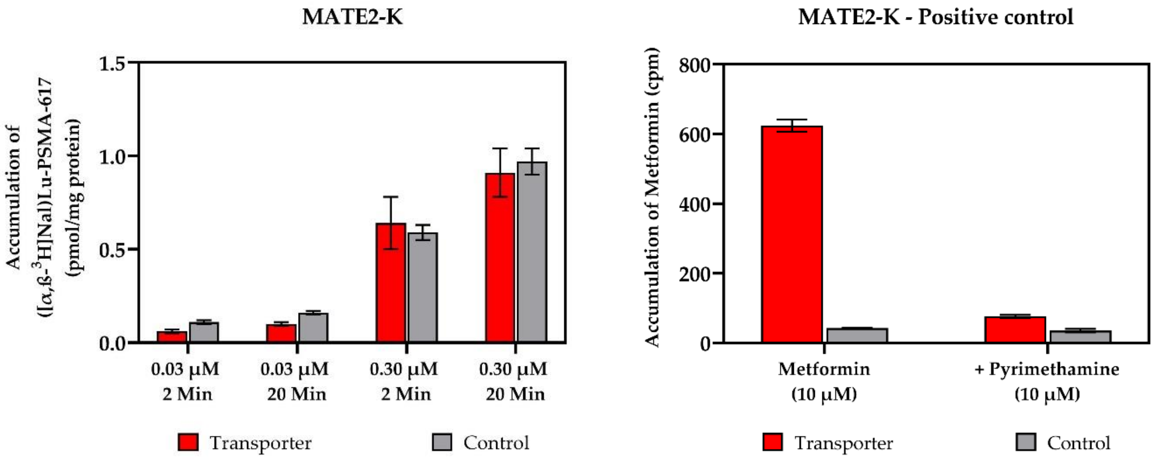

Figure S6: Graphical representation of ([α,β-^{3}H]Nal)Lu-PSMA-617-accumulation in MATE2-K transporter expressing (MDCKII-MATE2K-Fin) and control cells (MDCKII-CAT-Fin) applying 2- and 20-minute incubation periods, measured in SLC transporter substrate assay. Data are expressed as mean (n=3) ± SD. Concentrations are nominal.

Table S11: ([α,β-^{3}H]Nal)Lu-PSMA-617-accumulation in MATE2-K transporter-expressing (MDCKII-MATE2K-Fin) and control cells (MDCKII-CAT-Fin) applying 2- and 20-minute incubation periods, measured in SLC transporter substrate assay.

Compound	Conditions		Accumulation (pmol/mg protein)			Fold accumulation
	Nominal concentration [µM]	Minutes	In MATE2-K transporter expressing cells	In control cells	Active transport	
([α,β-^{3}H]Nal) Lu-PSMA-617	0.03	2	0.06 ± 0.01	0.11 ± 0.01	-0.05 ± 0.02	0.54
		20	0.10 ± 0.01	0.16 ± 0.01	-0.07 ± 0.01	0.59
	0.30	2	0.64 ± 0.14	0.59 ± 0.04	0.05 ± 0.15	1.08
		20	0.91 ± 0.13	0.97 ± 0.07	-0.06 ± 0.15	0.94
**Positive control (accumulation in cpm)**						
Metformin	10	5	624.33 ± 17.56	42.00 ± 1.00	582.33 ± 17.59	14.87
+ Pyrimethamine	+ 10		76.00 ± 4.36	35.33 ± 5.03	40.67 ± 6.66	2.15

Data are expressed as mean (n=3) ± SD.

4.3 Organic anion transporter OAT1

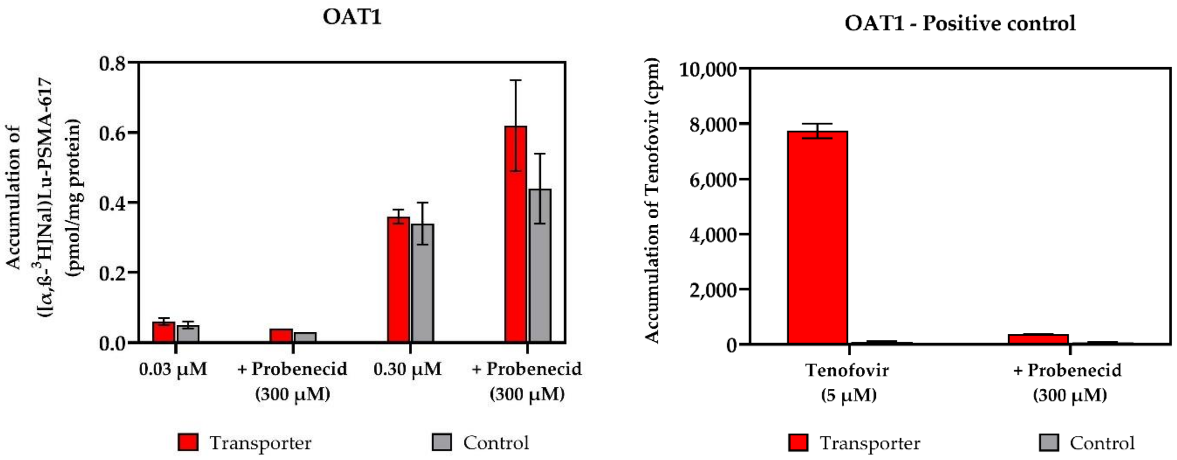

Figure S7: Graphical representation of ([α,β-^{3}H]Nal)Lu-PSMA-617-accumulation in OAT1 transporter expressing (HEK293-OAT1-LV) and control cells (HEK293-Mock-B-LV) applying 20-minute incubation periods, measured in SLC transporter substrate assay in the absence and presence of the reference inhibitor of OAT1 (Probenecid). Data are expressed as mean (n=3) ± SD. Concentrations are nominal.

Table S12: ([α,β-^{3}H]Nal)Lu-PSMA-617-accumulation in OAT1 transporter expressing (HEK293-OAT1-LV) and control cells (HEK293-Mock-B-LV) applying 20-minute incubation periods, measured in SLC transporter substrate assay in the absence and presence of the reference inhibitor of OAT1 (probenecid).

Compound	Conditions		Accumulation (pmol/mg protein)			Fold accumulation
	Nominal concentration [µM]	Minutes	In OAT1 transporter expressing cells	In control cells	Active transport	
([α,β-^{3}H]Nal) Lu-PSMA-617	0.03	20	0.06 ± 0.01	0.05 ± 0.01	0.01 ± 0.01	1.17
+ Probenecid	+ 300		0.04 ± 0.00	0.03 ± 0.00	0.00 ± 0.00	1.10
([α,β-^{3}H]Nal) Lu-PSMA-617	0.30	20	0.36 ± 0.02	0.34 ± 0.06	0.01 ± 0.06	1.04
+ Probenecid	+ 300		0.62 ± 0.13	0.44 ± 0.10	0.18 ± 0.16	1.42
**Positive control (accumulation in cpm)**						
Tenofovir	5	2	7739.67 ± 266.56	85.00 ± 24.27	7654.67 ± 267.66	91.05
+ Probenecid	+ 300		372.67 ± 12.58	77.00 ± 19.00	295.67 ± 22.79	4.84

Data are expressed as mean (n=3) ± SD.

4.4 Organic anion transporter OAT2v1

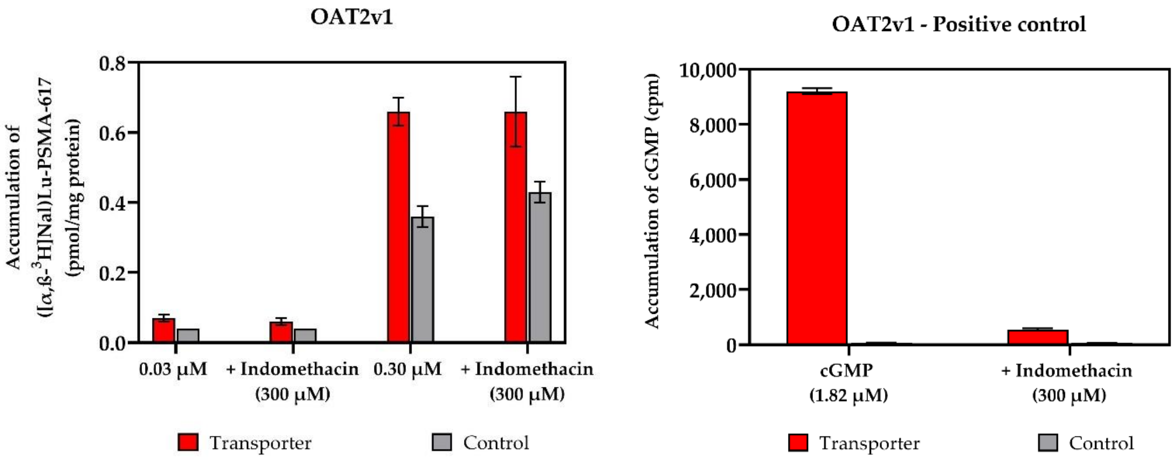

Figure S8: Graphical representation of ([α,β-^{3}H]Nal)Lu-PSMA-617-accumulation in OAT2v1 transporter expressing (HEK293-OAT2v1-LV) and control cells (HEK293-Mock-LV) applying 20-minute incubation periods, measured in SLC transporter substrate assay in the absence and presence of the reference inhibitor of OAT2v1 (Indomethacin). Data are expressed as mean (n=3) ± SD. Concentrations are nominal.

Table S13: ([α,β-^{3}H]Nal)Lu-PSMA-617-accumulation in OAT2v1 transporter expressing (HEK293-OAT2v1-LV) and control cells (HEK293-Mock-LV) applying 20-minute incubation periods, measured in SLC transporter substrate assay in the absence and presence of the reference inhibitor of OAT2v1 (indomethacin).

Compound	Conditions		Accumulation (pmol/mg protein)			Fold accumulation
	Nominal concentration [μM]	Minutes	In OAT2v1 transporter expressing cells	In control cells	Active transport	
([α,β-^{3}H]Nal) Lu-PSMA-617	0.03	20	0.07 ± 0.01	0.04 ± 0.00	0.03 ± 0.01	1.77
+ Indomethacin	+ 300		0.06 ± 0.01	0.04 ± 0.00	0.01 ± 0.01	1.24
([α,β-^{3}H]Nal) Lu-PSMA-617	0.30	20	0.66 ± 0.04	0.36 ± 0.03	0.30 ± 0.05	1.83
+ Indomethacin	+ 300		0.66 ± 0.10	0.43 ± 0.03	0.24 ± 0.10	1.55
**Positive control (accumulation in cpm)**						
cGMP	1.82	1	9207.33 ± 106.50	75.00 ± 2.65	9132.33 ± 106.53	122.76
+ Indomethacin	+ 300		551.67 ± 42.55	67.67 ± 5.51	484.00 ± 42.90	8.15

Data are expressed as mean (n=3) ± SD. cGMP: Guanosine cyclic phosphate.

4.5 Organic anion transporter OAT3

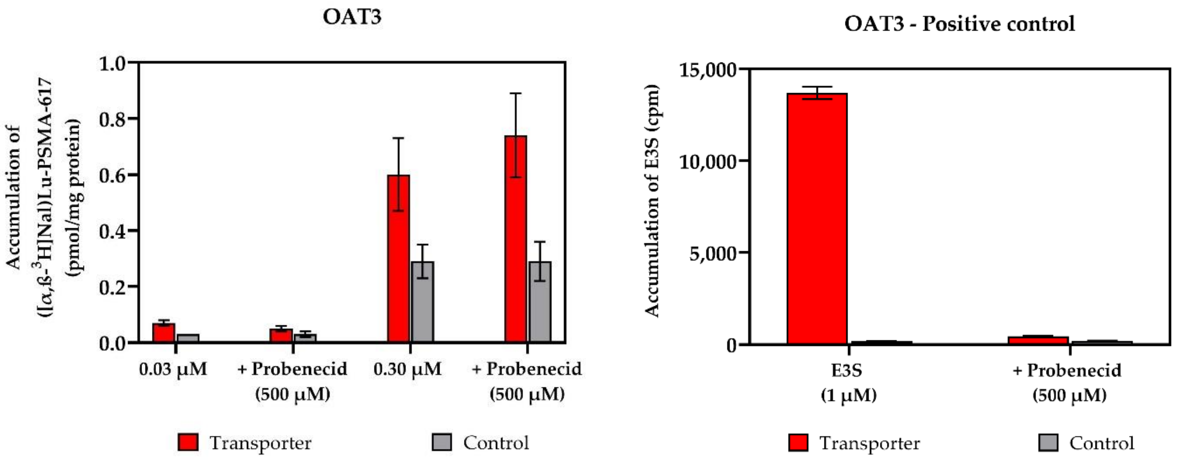

Figure S9: Graphical representation of ([α,β-^{3}H]Nal)Lu-PSMA-617-accumulation in OAT3 transporter expressing (HEK293-OAT3-LV) and control cells (HEK293-Mock-LV) applying 20-minute incubation periods, measured in SLC transporter substrate assay in the absence and presence of the reference inhibitor of OAT3 (Probenecid). Data are expressed as mean (n=3) ± SD. Concentrations are nominal.

Table S14: ([α,β-^{3}H]Nal)Lu-PSMA-617-accumulation in OAT3 transporter expressing (HEK293-OAT3-LV) and control cells (HEK293-Mock-LV) applying 20-minute incubation periods, measured in SLC transporter substrate assay in the absence and presence of the reference inhibitor of OAT3 (probenecid).

Compound	Conditions		Accumulation (pmol/mg protein)			Fold accumulation
	Nominal concentration [µM]	Minutes	In OAT3 transporter expressing cells	In control cells	Active transport	
([α,β-^{3}H]Nal) Lu-PSMA-617	0.03	20	0.07 ± 0.01	0.03 ± 0.00	0.03 ± 0.01	1.96
+ Probenecid	+ 500		0.05 ± 0.01	0.03 ± 0.01	0.02 ± 0.01	1.56
([α,β-^{3}H]Nal) Lu-PSMA-617	0.30	20	0.60 ± 0.13	0.29 ± 0.06	0.31 ± 0.14	2.09
+ Probenecid	+ 500		0.74 ± 0.15	0.29 ± 0.07	0.45 ± 0.17	2.52
**Positive control (accumulation in cpm)**						
E3S	1	1	13693.00 ± 337.98	182.33 ± 17.21	13510.67 ± 338.42	75.10
+ Probenecid	+ 500		454.67 ± 38.89	192.33 ± 28.99	262.33 ± 48.50	2.36

Data are expressed as mean (n=3) ± SD. E3S: Estrone-3-sulfate.

4.6 Organic anion transporter OAT4

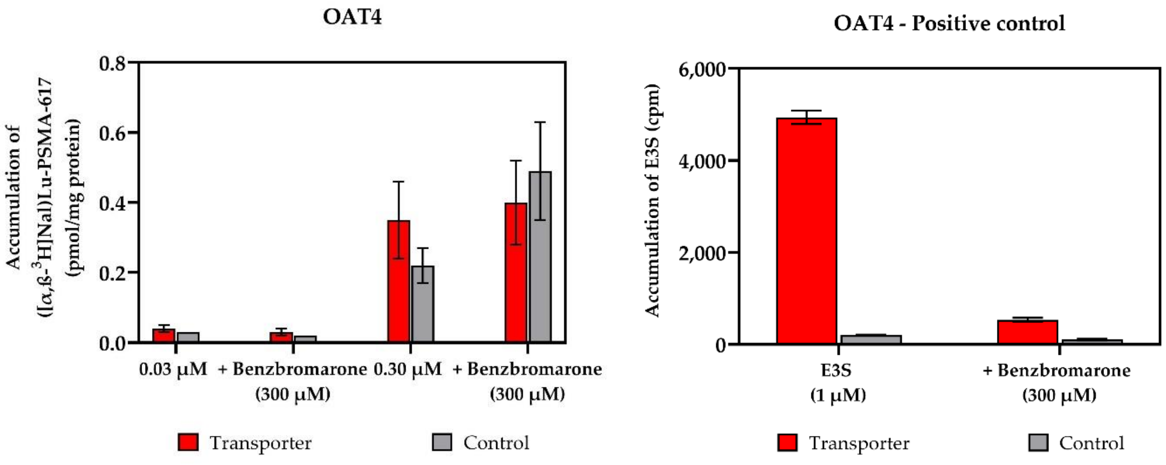

Figure S10: Graphical representation of ([α,β-^{3}H]Nal)Lu-PSMA-617-accumulation in OAT4 transporter expressing (HEK293-OAT4-LV) and control cells (HEK293-Mock-LV) applying 20-minute incubation periods, measured in SLC transporter substrate assay in the absence and presence of the reference inhibitor of OAT4 (Benzbromarone). Data are expressed as mean (n=3) ± SD. Concentrations are nominal.

Table S15: ([α,β-^{3}H]Nal)Lu-PSMA-617-accumulation in OAT4 transporter expressing (HEK293-OAT4-LV) and control cells (HEK293-Mock-LV) applying 20-minute incubation periods, measured in SLC transporter substrate assay in the absence and presence of the reference inhibitor of OAT4 (benzbromarone).

Compound	Conditions		Accumulation (pmol/mg protein)			Fold accumulation
	Nominal concentration [µM]	Minutes	In OAT4 transporter expressing cells	In control cells	Active transport	
([α,β-^{3}H]Nal) Lu-PSMA-617	0.03	20	0.04 ± 0.01	0.03 ± 0.00	0.01 ± 0.01	1.22
+ Benzbromarone	+ 300		0.03 ± 0.01	0.02 ± 0.00	0.01 ± 0.01	1.47
([α,β-^{3}H]Nal) Lu-PSMA-617	0.30	20	0.35 ± 0.11	0.22 ± 0.05	0.13 ± 0.12	1.58
+ Benzbromarone	+ 300		0.40 ± 0.12	0.49 ± 0.14	-0.09 ± 0.18	0.81
**Positive control (accumulation in cpm)**						
E3S	1	1	4936.67 ± 145.58	204.33 ± 8.96	4732.33 ± 145.86	24.16
+ Benzbromarone	+ 300		536.00 ± 42.33	104.67 ± 19.55	431.33 ± 46.63	5.12

Data are expressed as mean (n=3) ± SD. E3S: Estrone-3-sulfate.

4.7 Organic cation transporter OCT3

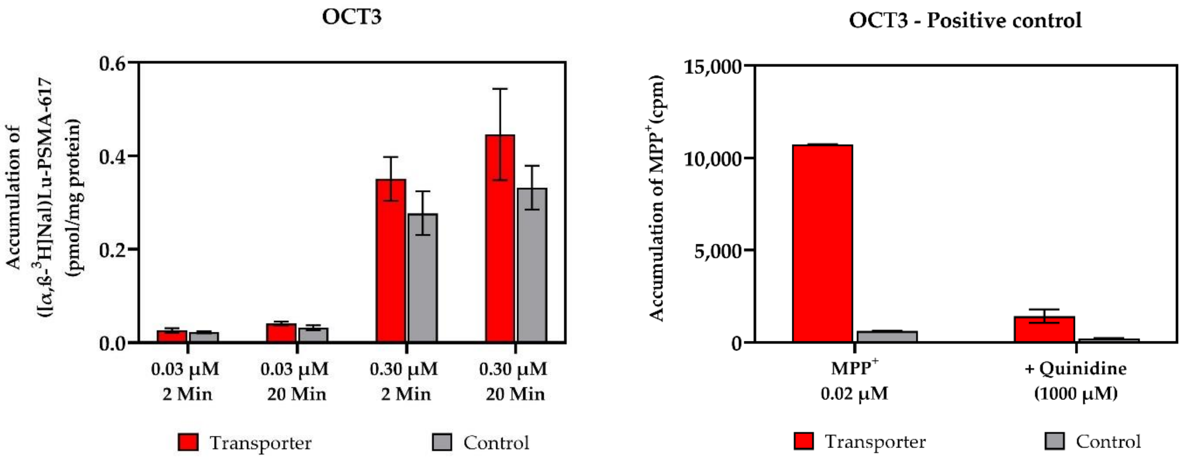

Figure S11: Graphical representation of ([α,β-^{3}H]Nal)Lu-PSMA-617-accumulation in OCT3 transporter expressing (HEK293-OCT3-LV) and control cells (HEK293-Mock-LV) applying 2- and 20-minute incubation periods, measured in SLC transporter substrate assay. Data are expressed as mean (n=3) ± SD. Concentrations are nominal.

Table S16: ([α,β-^{3}H]Nal)Lu-PSMA-617-accumulation in OCT3 transporter-expressing (HEK293-OCT3-LV) and control cells (HEK293-Mock-LV) applying 2- and 20-minute incubation periods, measured in SLC transporter substrate assay.

Compound	Conditions		Accumulation (pmol/mg protein)			Fold accumulation
	Nominal concentration [µM]	Minutes	In OCT3 transporter expressing cells	In control cells	Active transport	
([α,β-^{3}H]Nal) Lu-PSMA-617	0.03	2	0.026 ± 0.005	0.022 ± 0.002	0.003 ± 0.005	1.15
		20	0.041 ± 0.004	0.032 ± 0.005	0.009 ± 0.006	1.29
	0.30	2	0.351 ± 0.047	0.277 ± 0.047	0.074 ± 0.067	1.27
		20	0.446 ± 0.098	0.332 ± 0.047	0.113 ± 0.109	1.34
**Positive control (accumulation in cpm)**						
MPP$^+$	0.02	3	10728.333 ± 24.440	629.333 ± 3.786	10099.000 ± 24.732	17.05
+ Quinidine	+ 1000		1429.667 ± 364.256	233.000 ± 7.000	1196.667 ± 364.323	6.14

Data are expressed as mean (n=3) ± SD. MPP$^+$: 1-methyl-4-phenylpyridinium.

4.8 Peptide transporter PEPT2

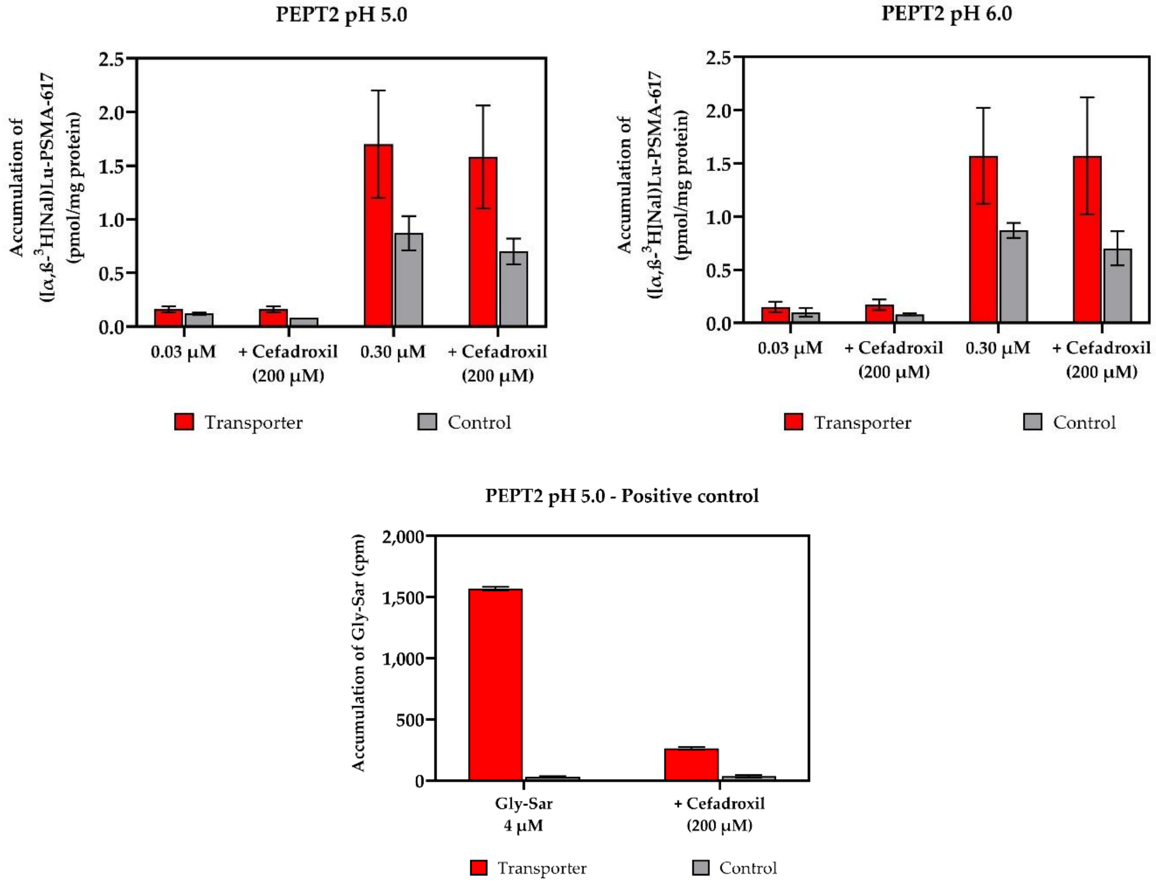

Figure S12: Graphical representation of ([α,β-^{3}H]Nal)Lu-PSMA-617-accumulation in PEPT2 transporter-expressing (CHO-K1-PEPT2) and control cells (CHO-K1) after 20-minute incubation period, measured in SLC transporter substrate assay in the absence and presence of the reference inhibitor of PEPT2 (Cefadroxil) at pH 5.0 and 6.0. Positive control conducted at pH 5.0. Concentrations are nominal.

Table S17: ([α,β-^{3}H]Nal)Lu-PSMA-617-accumulation in PEPT2 transporter-expressing (CHO-K1-PEPT2) and control cells (CHO-K1) after 20-minute incubation period, measured in SLC transporter substrate assay in the absence and presence of the reference inhibitor of PEPT2 (cefadroxil) at pH 5.0 and 6.0. Positive control conducted at pH 5.0.

Compound	Conditions (pH 5.0)		Accumulation (pmol/mg protein)			Fold accumulation
	Nominal concentration [μM]	Minutes	In PEPT2 transporter expressing cells	In control cells	Active transport	
([α,β-^{3}H]Nal) Lu-PSMA-617	0.03	20	0.16 ± 0.03	0.12 ± 0.01	0.04 ± 0.03	1.34
+ Cefadroxil	+ 200		0.16 ± 0.03	0.08 ± 0.00	0.08 ± 0.03	1.94
([α,β-^{3}H]Nal) Lu-PSMA-617	0.30	20	1.70 ± 0.50	0.87 ± 0.16	0.83 ± 0.53	1.95
+ Cefadroxil	+ 200		1.58 ± 0.48	0.70 ± 0.12	0.88 ± 0.50	2.27
**Compound**	**Conditions (pH 6.0)**		**Accumulation (pmol/mg protein)**			**Fold accumulation**
	**Nominal concentration [μM]**	**Minutes**	**In PEPT2 transporter expressing cells**	**In control cells**	**Active transport**	
([α,β-^{3}H]Nal) Lu-PSMA-617	0.03	20	0.15 ± 0.05	0.10 ± 0.04	0.06 ± 0.06	1.57
+ Cefadroxil	+ 200		0.17 ± 0.05	0.08 ± 0.01	0.09 ± 0.05	2.21
([α,β-^{3}H]Nal) Lu-PSMA-617	0.30	20	1.57 ± 0.45	0.87 ± 0.07	0.70 ± 0.45	1.81
+ Cefadroxil	+ 200		1.57 ± 0.55	0.70 ± 0.16	0.88 ± 0.58	2.26
**Positive control at pH 5.0 (accumulation in cpm)**						
Gly-Sar	4	8	1570.00 ± 15.59	31.33 ± 4.93	1538.67 ± 16.35	50.11
+ Cefadroxil	+ 200		263.67 ± 10.21	34.33 ± 11.55	229.33 ± 15.42	7.68

Data are expressed as mean (n=3) ± SD. Gly-Sar: Glycylsarcosine.

SI5. Treatment groups & controls of transporter substrate assays (SLC)

Treatment groups applied in the transporter substrate assays are listed in the table below:

Table S18: Treatment groups in SLC transporter substrate assays: MATE1, MATE2-K, OCT3.

Assessment of transporter-specific accumulation of TA	No. of wells
PSMA-617 at 0.03 and 0.30 μM (nominal) and two incubation time points (2 and 20 min) in triplicate in transporter expressing cells	12 per transporter
PSMA-617 at 0.03 and 0.30 μM (nominal) and two incubation time points (2 and 20 min) in triplicate in control cells	12 per transporter
Dosing solution samples	3 per concentration per transporter

Table S19: Treatment groups in SLC transporter substrate assays: OAT1, OAT2v1, OAT3, OAT4, PEPT2.

Assessment of transporter-specific accumulation of TA	No. of wells
PSMA-617 at 0.03 and 0.30 μM (nominal) and one incubation time point (20 min) in triplicate in transporter expressing cells in the absence or presence of the reference inhibitor	12 per transporter
PSMA-617 at 0.03 and 0.30 μM (nominal) and one incubation time point (20 min) in triplicate in control cells in the absence or presence of the reference inhibitor	12 per transporter
Dosing solution samples	3 per concentration per transporter

For controls:

- Uptake of the probe substrate in control cells provided background activity values for all data points.
- Incubation with probe substrate (solvent control) provided 100% activity values.
- A reference inhibitor served as a positive control for inhibition.
